# Clinical Attributes and Electroencephalogram Analysis of Patients With Varying Alpers’ Syndrome Genotypes

**DOI:** 10.3389/fphar.2021.669516

**Published:** 2021-10-06

**Authors:** Hua Li, Wei Wang, Xiaodi Han, Yujia Zhang, Lifang Dai, Manting Xu, Jie Deng, Changhong Ding, Xiaohui Wang, Chunhong Chen, Xiaofeng Yang, Fang Fang

**Affiliations:** ^1^ Department of Neurology, Beijing Children’s Hospital, Capital Medical University, National Center For Children’s Health, Beijing, China; ^2^ Laboratory of Brain Disorders, Ministry of Science and Technology, Collaborative Innovation Center for Brain Disorders, Beijing Institute of Brain Disorders, Capital Medical University, Beijing, China; ^3^ Bioland Laboratory Guangzhou Regenerative Medicine and Health Guangdong Laboratory, Guangzhou, China

**Keywords:** Alpers, syndrome, POLG, electroencephalogram, RHADS, diagnosis

## Abstract

Alpers’ syndrome is an early inceptive neurodegenerative disorder with a poor prognosis, characterized by developmental regression, intractable epilepsy, and hepatic dysfunction. Candidate genes, such as *POLG, PARS2, CARS2, FARS2, NARS2, and GABRB2* are distinguished and registered following research on large cohorts that portray the clinical phenotype in such patients using expanded access to whole-exome sequencing (WES). In this study, we aimed to better understand the electroencephalogram (EEG) characteristics and clinical phenotype of different genotypes of the Alpers’ syndrome, which are currently insufficiently studied. We conducted a study on seven patients with Alpers’ syndrome who received treatment in Beijing Children’s Hospital and had a detailed clinical EEG. Furthermore, a substantial literature search of the Chinese Biomedical Literature Database, PubMed, and Cochrane Central Register of Controlled Trials EMBASE was also conducted, which revealed a total of 22 reported cases between January 2008 to January 2021. We analyzed 29 cases of Alpers’ syndrome caused by different gene variants, of which 22 cases were related to *POLG* gene mutation and 7 cases were related to *PARS2, CARS2, FARS2, NARS2, and GABRB2* gene mutation, and found that patients with distinctive pathogenic variants exhibited comparable phenotypes and similar EEG patterns. And we defined EEG characteristics found specifically in Alpers’ syndrome. Rhythmic high-amplitude delta with superimposed (poly) spikes (RHADS) is a characteristic EEG finding in the early stages of Alpers’ syndrome and is a kind of epileptic phenomenon, which can provide clues for the early diagnosis of the disease.

## Introduction

Alpers’ syndrome is an early inceptive neurodegenerative disorder with a poor prognosis, characterized by the triad of developmental regression, intractable epilepsy, and hepatic dysfunction (Sofou et al., 2012; Tarka et al., 2020). The patients with Alpers’ syndrome are generally less than 4 years old after the initial presentation of symptoms (Hannah and Yasir, 2021). It usually occurs in infancy or early childhood, and rarely in juveniles. In the early stages of the disease, epileptic seizures, as an early symptom, are the most common. The epileptic seizures develop in the form of focal, multifocal or myoclonic seizures evolving into epilepsia partialis continua (EPC) or myoclonic status epilepticus (MSE). The sudden outbreak of therapeutically refractory seizures in infancy or adolescence is considered an average clinical indicator of Alpers’ syndrome. The electroencephalograms (EEG) finds have shown a specific EEG phenomenon, known as rhythmic high-amplitude delta with superimposed (poly) spikes (RHADS) (Wolf et al., 2009; van Westrhenen et al., 2018). The epileptic seizures may be have an evolution of EEG in the development of Alpers’ syndrome (Hannah and Yasir, 2021). EEG might provide in the potential diagnostic clue (Wolf et al., 2009; van Westrhenen et al., 2018).

Recent studies have identified molecular genetic causes of Alpers’ syndrome, including pathogenic mutations in the gene encoding the catalytic subunit of polymerase gamma (*POLG*), *PARS2* which encodes prolyl-tRNA synthetase, *FARS2* which encodes phenylalanyl-tRNA synthetase, and *NARS2* which encodes asparaginyl-tRNA synthetase, etc, *POLG* gene variation is the most common (Sofou et al., 2015; Walker et al., 2016; Samanta et al., 2018; Sofou et al., 2021).

Alpers’ syndrome is one of the most serious phenotypes of mitochondrial disease caused by *POLG* gene mutation. More than 90% of Alpers’ syndrome cases happen due to autosomal recessive mutations in the nuclear-encoded catalytic subunit (Bicknese et al., 1992; Hance et al., 2005; Chrysostomou et al., 2016). The results of *POLG* flaws are mtDNA depletion/accumulation of mtDNA deletions. The depletion of mtDNA causes consequent cellular dysfunction, mitochondrial respiratory chain impairments, and apoptosis in the brain and liver (Anagnostou et al., 2016; Naviaux RK et al., 1999; Tzoulis C et al., 2014).

With the development of genetic detection and analysis techniques, the pathogenic genes of Alpers’ syndrome have been discovered. The candidate genes such as *POLG, PARS2, CARS2, FARS2, NARS2,* and *GABRB2* have been distinguished and registered using large cohorts that portray the clinical phenotype in such patients using expanded access to WES. However, at present, no systematic studies have been performed on the clinical phenotypes and the EEG findings that occur in patients with different genetic pathogenic variation mutations. In this study, we aimed to characterize the EEG features and clinical phenotypes of patients with different genotypes of the Alpers’ syndrome and assist in future studies.

## Methods

### Patients

This study was conducted on seven patients with Alpers’ syndrome who underwent a thorough medical treatment from May 2013 to December 2020 in the Beijing Children’s Hospital. They were examined for detailed clinical, EEG, and pathogenic information.

In addition to these, a substantial literature search was carried out in EMBASE, Cochrane Central Register of Controlled Trials, and PubMed. Prior to an extensive literature search, an eligibility criterion was taken into consideration. It included EEG case studies based on patient’s medical history with Alpers’ syndrome. Publications in any language other than English, anthropoidal studies, limited resources of literature review, and unauthenticated abstracts were considered as criteria for exclusion, and comprehensive analysis was reported. After applying the inclusion and exclusion criteria, we shortlisted 22 cases that were reported in the various databases from January 2008 to January 2021. Thus, we examined a total of 29 patients whose details may be found in Supplementary Table S1.

The clinical features that were evaluated for these 29 patients with Alpers’ syndrome were as follows: sex of the patient, age at disease onset, age at seizure onset, categories of seizures, seizure types at disease onset, development, EEG, pathogenic information, and other clinical features. Seizures and epilepsy syndromes were categorized as per the rules and regulations of the International League Against Epilepsy (ILAE) (Belousova et al., 2017).

### Genetic Analysis

In the present study, all samples were captured by whole-exome reagent hybridization and using Illumina instruments for next-generation sequencing. Sequence data aligned with the human genome reference (hg19) and variants-calling were carried out with NextGene V2.3.4 software (Softgenetics LLC., State College, PA, United States). Variants were screened as follows:1) preference to the disease-related variants, small insertions and deletions (INDEL), canonical splice sites, and nonsense variants; 2) minor allele frequency (MAF) in normal populations <5% (except for known MAF ≥5% pathogenicity); 3) preference to variants in the Human Gene Mutation Database (HGMD), ClinVar; and 4) preference to variants in the Online Mendelian Inheritance in Man database. Pathogenic variants were defined according to the standards and guidelines for the interpretation of sequence variants published by the American College of Medical Genetics in 2015 with Human Genome Variation Society nomenclature (Richards et al., 2015). We performed Sanger sequencing of all samples to validate the identified *POLG* mutations and test the parental origin of available family members. Of these, we also used quantitative PCR technology to verify the deletion of large fragments of the genome in ample No. 6.

### EEG Recording and Analysis

Video-EEG was documented and digitized at the sample rate of 1000 Hz using the standard international 10–20 system (Natus Medical Incorporated, Pleasanton, CA, United States). Impedances were kept below 10 KΩ. A low-cut filter at 1 Hz was used before digital sampling. At least a prolonged continuous EEG monitoring (more than 24-h-long video-EEG recording). All EEG data were interpreted and reviewed by qualified neurophysiologists adopting the following criteria for RHADS: 1) occipital strength; 2) a moderate movement (<1 Hz) of high amplitude (200–1,000 uV); 3) frequent occurrence; and 4) a set of superimposed polyspikes. EEG data that showed results as ‘inexplicit’ were excluded for additional analysis.

In EEGs with RHADS, gamma oscillations (30–80 Hz) were marked visually via time-frequency analyses for EEG. First, the EEG data were reviewed visually in bipolar 10–20-Montage, and stage-Ⅱ NREM sleep data (the delta band was estimated higher than 25% of all delta bands in a 30-s epoch by visual inspection) was selected (Bagshaw et al., 2009; Jacobs et al., 2016). Then, after band-pass filtering the raw data (30–80 Hz), time-frequency analysis techniques were used in each segment of data. Finally, the gamma oscillations were identified and compared with the original EEG data. The EEG data were visually examined by two EEG professional specialists, and channels with significant artifacts were excluded from the analysis.

### Statistical Analysis

A total of 29 data samples were collected in this study, including 22 cases in the *POLG* group and 7 cases in the NON-*POLG* group. The differences of various indicators in the two groups were compared. For continuous variables that did not conform to the normal distribution, the median description was adopted, and a non-parametric test was used to compare the differences. For the comparison of classification variables, the composition ratio description was adopted, and the differences were compared by Fisher exact test was used to compare proportions of patients with microcephaly, Infantile spasm, Hepatic dysfunction, and EEG feature. For survival analysis, the endpoint was time to death which was defined as the time in months from the date of disease onset to the date of death. The survival rate of the two groups was compared by Kaplan-Meier survival curve analysis. The software selected for this analysis was SPSS26 and Graphpad7.0, and the significance level was 0.05.

### Ethics Statement

The present study has been approved by the Ethics Committee of Beijing Children’s Hospital and written informed consent was obtained from the guardians/families of the patients for their participation in the study.

## Result

### Mutation Outcomes

**FIGURE 1:**
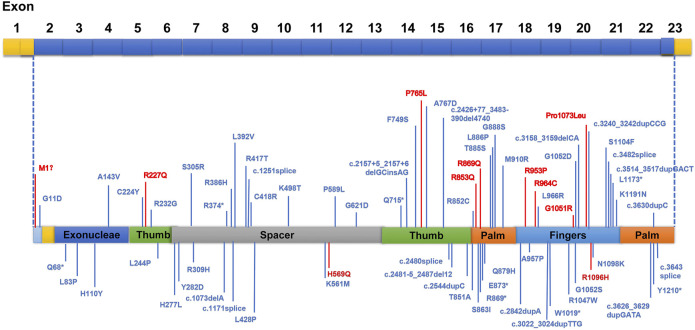
Areas of distinguished variants in *POLG* mutation in Alpers’ syndrome. Mutations presented in red were identified in this current study, whereas those in blue have been reported by past investigations.

Our study revealed a total of 12 different variations within the *POLG* gene in seven patients (patient 1 to 5, previously reported (Dai et al., 2019) (Figure 1). 10 mutations were reported and two mutations were novel (Table 1, patient 6: ex.2-23del and patient 7: p. H569N). Patient 6 was compound heterozygous for the c.3218C>T mutation and a novel variation, a deletion in exon 2–23 (chr15:89859622–89876990). Quantitative PCR technology was used to verified the deletion, the proband and his mother were heterozygous deletions.

**TABLE 1:** Summary of the Genetic mutation variants of 29 cases with Alpers’ syndrome.

Patient	Ref	Gene	Nucleotide	Protein	Parental origin	ACMG-guideline	ACMG/hgmd CLASS	Mutation Taster	Polyphen-2_HVAR	SIFT
1	Present case	*POLG* (NM_002693.2)	c.2858G > C	p.R953P	paternal	PM1 + PM5 + PM2_Supporting + PP3	LP	DC	PD	D
			c.2294C > T	p.P765L	maternal	PM1 + PM5 + PM2_Supporting + PP3	LP	DC	PD	D
2	Present case	*POLG* (NM_002693.2)	c.3151G > A	p.G1051R	maternal	PM1 + PM5 + PM2_Supporting + PP3	LP	DC	PD	D
			c.2606G > A	p.R869Q	paternal	PM3_Strong + PM1 + PM2_Supporting + PP3	VUS	DC	PD	D
3	Present case	*POLG* (NM_002693.2)	c.680G > A	p.R227Q	maternal	PM3 + PM5_Strong + PM2_Supporting + PP3	LP	DC	PD	D
			c.2T > G	p.M1?	paternal	PVS1_Supporting + PM3 + PM2_Supporting	VUS	DC	PD	D
4	Present case	*POLG* (NM_002693.2)	c.2558G > A	p.R853Q	de novo*	PM1 + PM3 + PM5 + PM2_Supporting + PP3	P	DC	PD	D
			c.2890C > T	p.R964C	maternal	PM1 + PM3_Strong + PM2_Supporting + PS3_Spporting + PP1 + PP3 + PP4	VUS	DC	PD	D
5	Present case	*POLG* (NM_002693.2)	c.3218C > T	p.P1073L	paternal	PM3_Strong + PS3_Moderate + PM2_Supporting + PP3 + PP4	P	DC	PD	D
			c.3218C > T	p.P1073L	maternal	PM3_Strong + PS3_Moderate + PM2_Supporting + PP3 + PP4	P	DC	PD	D
6	Present case	*POLG* (NM_002693.2)	c.3218C > T	p.P1073L	paternal	PM3_Strong + PS3_Moderate + PM2_Supporting + PP3 + PP4	P	DC	PD	D
			ex.2-23del	p.?	maternal	PVS1 + PM3 + PM2_Supporting	LP	NA	NA	NA
7	Present case	*POLG* (NM_002693.2)	c.1705C > A	p.H569N	maternal	PM3 + PM5 + PM2_Supporting + PP3	VUS	DC	PD	D
			c.3287G > A	p.R1096H	paternal	PM5_Strong + PM3 + PM2_Supporting + PP3 + PS3_Supporting	LP	DC	PD	D
8	Wiltshire et al. (2008)	*POLG* (NM_002693.3)	c.2551A > G	p.T851A	NA	DM	DM	NA	NA	NA
			c.3139C > T	p.R1047W	NA	DM	DM	NA	NA	NA
9	Wolf et al. (2009)	*POLG* (NM_002693.3)	c.2243G > C	p.W748S	NA	DM	DM	NA	NA	NA
			c.2542G > A	p.G848S	NA	DM	DM	NA	NA	NA
10	Wolf et al. (2009)	*POLG* (NM_002693.3)	c.2243G > C	p.W748S	NA	DM	DM	NA	NA	NA
			c.2542G > A	p.G848S	NA	DM	DM	NA	NA	NA
11	Wolf et al. (2009)	*POLG* (NM_002693.3)	c.1399G > A	p.A467T	NA	DM	DM	NA	NA	NA
							
12	Wolf et al. (2009)	*POLG* (NM_002693.3)	c.2243G > C	p.W748S	NA	DM	DM	NA	NA	NA
			novel splice mutation	novel splice mutation	NA	NA	NA	NA	NA	NA
13	Wolf et al. (2009)	*POLG* (NM_002693.3)	c.1399G > A	p.A467T	NA	DM	DM	NA	NA	NA
							
14	Uusimaa et al. (2008)	*POLG* (NM_002693.3)	c.2243G > C	p.W748S	NA	DM	DM	NA	NA	NA
			c.3428A > G	p.E1143G	NA	DFP	DFP	NA	NA	NA
15	Uusimaa et al. (2008)	*POLG* (NM_002693.3)	c.2243G > C	p.W748S	NA	DM	DM	NA	NA	NA
			c.3428A > G	p.E1143G	NA	DFP	DFP	NA	NA	NA
16	Uusimaa et al. (2008)	*POLG* (NM_002693.3)	c.2243G > C	p.W748S	NA	NA	DM	NA	NA	NA
			c.3428A > G	p.E1143G	NA	DFP	DFP	NA	NA	NA
17	Mccoy et al. (2011)	*POLG* (NM_002693.3)	c.1681G > A	p.A467T	NA	NA	NA	NA	NA	NA
			c.2897T > G	p.L966R	NA	DM	NA	NA	NA	NA
18	Mccoy et al. (2011)	*POLG*	c.2824G > A	p.A467T	NA	NA	NA	NA	NA	NA
		*POLG* (NM_002693.3)	c.2542G > A	p.G848S	NA	DM	NA	NA	NA	NA
19	Mccoy et al. (2011)	*POLG*	c.2836C > T	p.A467T	NA	NA	NA	NA	NA	NA
		*POLG* (NM_002693.3)	c.2554C > T	p.R852C	NA	DM	NA	NA	NA	NA
20	Mccoy et al. (2011)	*POLG* (NM_002693.3)	c.1399G > A	p.A467T	NA	DM	NA	NA	NA	NA
			c.1252T > C	p.C418R	NA	DM	NA	NA	NA	NA
21	Allen et al. (2014)	*POLG* (NM_002693.3)	c.2836C > T	p.A467T	NA	NA	NA	NA	NA	NA
			c.2740A > C	p.T914P	NA	DM	NA	NA	NA	NA
22	London et al. (2017)	*POLG* (NM_002693.3)	c.2836C > T	p.A467T	NA	NA	NA	NA	NA	NA
			c.2243G > C	p.W748S	NA	DM	NA	NA	NA	NA
23	sofou et al. (2015)	NARS2 (NM_024678.6)	c.641C > T	p.P214L	NA	NA	NA	NA	NA	NA
24	sofou et al. (2015)	PARS2 (NM_152,268.4)	c.1130dupC	p.K328fs*1	NA	DM	NA	NA	NA	NA
					NA	NA	NA	NA	NA	NA
25	Samanta et al. (2018)	CARS2 (NM_024537.4)	c.155T > G	p.V52G	NA	DM	NA	NA	NA	NA
			c.563C > T	p.T188M	NA	DM	NA	NA	NA	NA
26	Nishikawa et al. (2020)	GABRB2 (NM_021911.3)	c.784G > T	p.V262P	NA	DM	NA	NA	NA	NA
27	sofou et al. (2021)	NARS2 (NM_024678.6)	c.641C > T	p.P214L	NA	DM	NA	NA	NA	NA
28	sofou et al. (2021)	NARS2 (NM_024678.6)	c.641C > T	p.P214L	NA	DM	NA	NA	NA	NA

29	Walker et al. (2016)	FARS2 (NM_006567.5)	c.253C > G	p.P85A	NA	DM	NA	NA	NA	NA
			c.403C > G	p.H135D	NA	DM	NA	NA	NA	NA

Abbreviations: LP, likely pathogenic; P, pathogenic; VUS, uncertain significance; DC, disease causing; NA, not available; PD, probably damaging; D, damaging; DM, disease-causing mutation; DFP, disease-associated polymorphism with supporting functional evidence.

* we not verified that weather patient’father is chimerism or not biological father due to ethical and sample acquisition reasons.

We identified pathogenic/likely pathogenic/VUS variants in seven patients in our cohort. One patient (patient5) in our study shared the same homozygous variant c.3218C>T (p.P1073L), which was also reported in another two Chinese studies (Qian et al., 2015). Three different recurrent variants (p.W748S,p.E1143G, and p. A467T) were reported (Uusimaa et al., 2008; Wolf et al., 2009). Two unrelated patients shared the same recurrent homozygous variant c.3218C>T (p.P1073L), this recommends a foundation effect in the Chinese population. The other five patients harbored compound heterozygous variants, including 2 variants (Exon 2–23 del and c.1705C>A,p.H569N) newly reported in this study (Table 1).

Genetic (the classification of the variants according to the American College of Medical Genetics and Genomics (ACMG), parental origin, etc) details of these patients are shown in Table 1. Among the 29 Alpers’ syndrome patients, 79.31% (23/29) carried at least 1 missense variant. Only 1 truncation variant in *POLG* was identified (Wolf et al., 2009). Variants in *PARS2*, *CARS2*, *GABRB2*, *FARS2,* and *NARS2* were also reported in Alpers’ syndrome patients (Sofou et al., 2015; Walker et al., 2016; Samanta et al., 2018; Sofou et al., 2021). All the variations were consistent with the autosomal recessive inheritance pattern, except for that in *GABRB2.* Aiko et al. reported a *de novo* missense variant in *GABRB2* (c.784G>T (p.Val262Phe)). A recurrent missense variant, c.641C>T (p.P214L), was identified in *NARS2* and 1 truncation variant was identified in *PARS2* (Sofou et al., 2015). All together 5 recurrent missense variants in 2 genes were identified in Alpers’ syndrome patients, and only 2 truncation variants were observed in 2 genes.

### Clinical Characteristics of Patients With Pathogenic *POLG* Variations

Our data showed that patients with pathogenic *POLG* variations showed the characteristics of mitochondrial encephalopathy, with refractory epilepsy, progressive liver function impairment, as the main clinical symptoms, acute liver failure occured after valproic acid application. For the analysis of *POLG*-related Alpers’ syndrome, 22 out of 29 patients were included (8 females, 14 males, The median age at onset was 2.33 years, the range of age-onset was from 2 months to 17 years). The clinical characteristics of *POLG* mutation-related Alpers’ syndrome were analyzed as follows.

### Seizures

All 22 patients (100%) with *POLG* mutation-related Alpers’ syndrome experienced epileptic seizures. A variety of refractory epileptic seizures were exhibited in 21 patients (95.5%). Seizures were the most common initial symptom (63.6%), and began at a median age of 2.67 years (ranging from 3 months to 17 years). The main type of seizures were focal/myoclonic seizures that lead to focal motor status or epileptic status. A total of 14 (63.6%) patients showed the symptoms of focal seizures, 12 (54.5%) patients had focal motor status or epileptic status, whereas myoclonic seizures occurred in 3 of 22 patients (13.6%) and generalized tonic-clonic seizures occurred in 2 of 22 patients (9.1%). Only 1 (4.5%) patient presented with the symptoms of generalized seizure. Eight of the 22 patients presented with the symptoms of epilepsia partialis continua (EPC; 36.4%) (Supplementary Table S1).

### Development

A notable developmental delay with the signs of *POLG* mutation was found in all patients who suffered from Alpers’ syndrome in life. A total of 6 out of 22 patients (27.3%) showed the symptoms of developmental delay that were evident before the onset of seizures. Nine patients exhibited normal development until the beginning of intractable seizures. One patient who was mentioned in the literature review was having the symptoms of severe-to-profound intellectual disability.

### Other Features

A series of features such as visual symptoms and visual disturbance, ataxia (including congenital ataxia), hypotonia, vomiting, language delayed/lost speech, and different levels of liver failure were found in all patients. Four patients had severe liver failure after valproic acid application and one patient showed multiorgan failure.

### Clinical Characteristics of Patients Without Pathogenic *POLG* Variants

In addition, Our data showed that patients with pathogenic NON-*POLG* (*NARS2, FARS2, PARS2, CARS2*, and *GABRB2*) variations showed the characteristics of age at onset was earlier, mostly in the early infantile period, with prominent infantile spasms, less liver damage, and microcephaly, most of the survival time after treatment is longer. The characteristics are significantly different from *POLG*-related Alpers’ syndrome. For the analysis of NON-*POLG* mutation-related Alpers’ syndrome, 7 out of 29 patients were included (2 females, 5 males, The age at onset was the early infantile period). The clinical characteristics of NON-*POLG* mutation-related Alpers’ syndrome were analyzed as follows.

### Seizures

All 7 patients (100%) with NON-*POLG* mutation-related Alpers’ syndrome experienced epileptic seizures. A variety of refractory epileptic seizures were exhibited in 7 patients (100%). Epileptic seizures were initial symptom in 3 patients. In terms of seizure type, two (43.1%) patients showed the symptoms of focal seizures, two (28.6%) exhibited focal motor status or epileptic status, whereas myoclonic seizures occurred in three of the seven patients (42.9%), generalized tonic-clonic seizures in three of the seven patients (42.9%), myoclonic seizures in 3 of 7 patients (42.9%). Only one (14.3%) patient presented with the symptoms of infantile spasm, one (14.3%) with the symptoms of atypical absence seizure, one (14.3%) with the symptoms of a tonic seizure, and three with the symptoms of EPC.

### Development

A notable developmental delay with signs of NON-*POLG* mutation was found in six of the seven patients who suffered from Alpers’ syndrome in life. Five out of seven patients (71.4%) showed symptoms of developmental delay that were evident before the seizure outbreak. One patient in the literature review showed symptoms of severe intellectual disability. Only one patient exhibited normal development until the beginning of intractable seizures.

### Other Features

The other series of features such as hypotonia (85.7%, 6/7), visual symptoms (42.9%, 3/7), feeding difficulties, vomiting (42.9%, 3/7), progressive microcephaly (42.9%,3/7), language delayed/lost speech (14.3%, 1/7), mild liver failure (28.6%, 2/7), and autism spectrum (14.3%, 1/7) were observed in the patients in this category. A detailed description of the clinical features of each patient is provided in Supplementary Table S1.

### EEG Results

**FIGURE 2:**
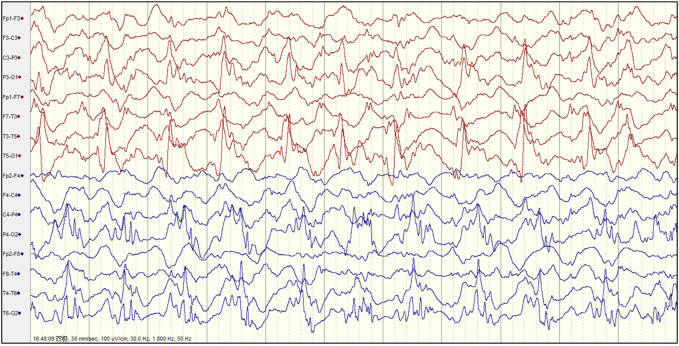
The electroencephalogram (EEG) of patient 4 with Alpers’ syndrome in our study showed typical rhythmic high amplitude delta waves with superimposed (poly) spikes (RHADS) in the bilateral posterior head region.

**FIGURE 3:**
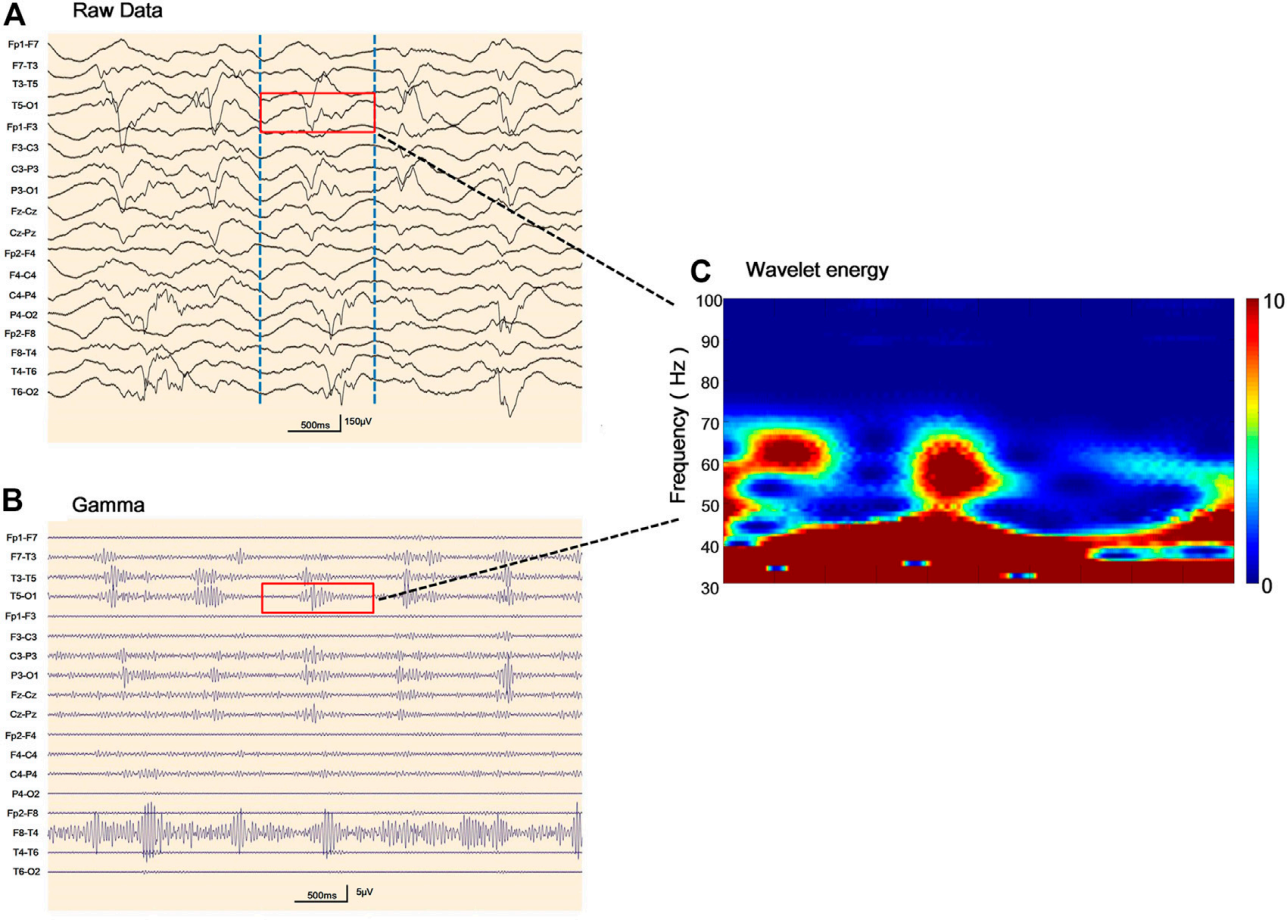
Representative electroencephalogram (EEG) follow-ups and corresponding gamma oscillations **(A)** Raw EEG with the pattern of rhythmic high amplitude delta waves with superimposed (poly) spikes (RHADS) in sleep during stage II non-rapid eye movement period, the red layout marks 1 s data **(B)** EEG corresponding to Figure 3A after 30–80 Hz filtering, the red line addresses the gamma oscillations visually identified **(C)** Verification of gamma oscillations. Utilizing the wavelet transform method, the time-frequency spectra exhibit spectral blobs at around 30–80 Hz in the region T5-O1 in association with the corresponding spikes.

Abnormal EEGs with epileptiform discharges and slow activity were detected at the early stage in all patients. A total of 23 out of 29 (79.3%) patients showed a reducing nonspecific background in the EEG. Of the 29 patients, focal epileptiform discharges on EEG were found in 16 patients (55.2%), multifocal epileptiform discharges were found in 2 (6.9%), and hypsarrhythmia occurred only in 1 (3.4%). Focal slowing and epileptiform discharges were seen relatively equally in the early and late stages of the disease. The incidence of RHADS was described in 18 out of 29 patients (62.1%) (Figure 2). RHADS were the most common EEG patterns and were based on focal abnormality along with epileptic discharges, and were described in four patients in our series with the gamma oscillations (Figure 3). RHADS and slowing were the most common factors identified in the occipital region (57.1%), followed by the impairment in the frontal lobe (28.6%) and temporal (14.3%) regions. In the late stage of the disease, six cases of RHADS on EEG performed focal changes (focal slowing, epileptiform discharges), multifocal epileptiform discharges, and burst suppression pattern.

### A Comparative Relation Between Genetic and Clinical Phenotypes of Patients With Alpers^,^ Syndrome

**FIGURE 4:**
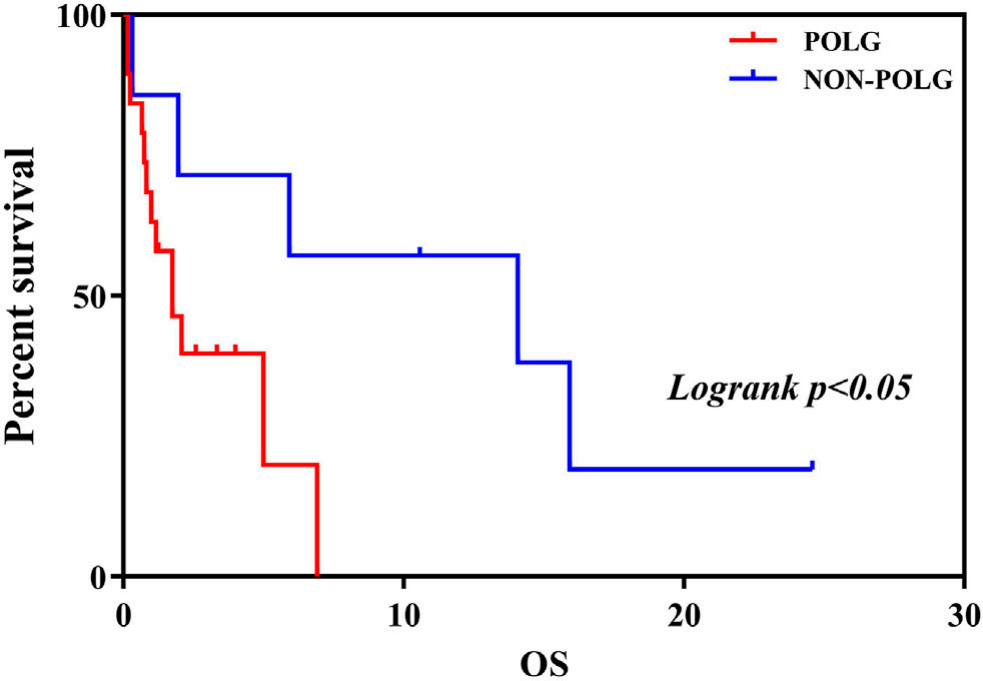
Kaplan-Meier curve comparing survival of patients with the *POLG* or non-*POLG* group and patients with *POLG* Group showed carried significantly worse survival.

As demonstrated in Table 2, Age at onset, Age at seizure onset, Time of death showed a significant relationship with the *POLG* or NON*-POLG* group,*p* < 0.05; As demonstrated in Table 2, Age at onset, Age at seizure onset, Time of death showed a significant relationship with the *POLG or* NON*-POLG* group,*p* < 0.05; microcephaly, Infantile spasm, Hepaticdys function, EEG feature showed a significant relationship in *POLG* or NON*-POLG* group, *p* < 0.05 (Table 3); As can be seen from the above table, there was a significant difference in survival rate between the NON*-POLG* group and the *POLG* group (*p* < 0.05). From the above table, there are significant differences in survival between NON*-POLG* group and *POLG*, *p* < 0.05. Among them, the median survival of Group *POLG* was 1.75 years and NON*-POLG* 14.083, and the survival rate of Group NON*-POLG* was signnificantly higher than that of Group *POLG*, with the following KM survival curve (Figure 4).

**TABLE 2:** Comparison of mutations and clinical features of 29 cases with Alpers’ syndrome by nonparametric test.

	Mutation involved		Z	P
	*POLG*	NON-*POLG*		
Age at onset	2.33 (0.65∼9.6)	0.21 (0.08∼0.92)	−2.271	0.023
Age at seizure onset	2.67 (0.83∼9.6)	0.5 (0.33∼8)	−1.963	0.049
Age at death	2.67 (1∼11.5)	13.33 (4.08∼15.5)	−1.487	0.137
Time of death	0.83 (0.17∼1.75)	5.92 (1.15∼15)	−2.053	0.04

**TABLE 3:** Comparison of mutations and clinical features of 29 cases with Alpers, syndrome by Fisher exact test.

		Mutation involved		p
		POLG	NON-POLG	
sex	M	8 (36.4%)	5 (71.4%)	0.192
	F	14 (63.6%)	2 (28.6%)	
microcephaly	no	22 (100%)	4 (57.1%)	0.01
	yes	0 (0%)	3 (42.9%)	
Infantile spasm	no	22 (100%)	4 (57.1%)	0.01
	yes	0 (0%)	3 (42.9%)	
Hepatic dysfunction	no	6 (27.3%)	6 (85.7%)	0.011
	yes	16 (72.7%)	1 (14.3%)	
EEG feature	no	6 (27.3%)	6 (85.7%)	0.011
	yes	16 (72.7%)	1 (14.3%)	

## Discussion

Alpers’ syndrome is a rare mitochondrial encephalopathy, which is thought to be caused by autosomal recessive mutations in the nuclear-encoded catalytic subunit of *POLG* (Hannah and Yasir, 2021). Pathogenic variants in *POLG* are common causes of multiple mtDNA deletions and/or mtDNA depletion. The brain and liver are susceptible to the disease because of their high demand for energy and proportionate need for mitochondria. The loss of mitochondria in these organ systems can lead to various symptoms, the most common being seizures and liver failure. Mitochondrial diseases affect cellular energy production through oxidative phosphorylation (OxPHOS) (Dimauro et al., 2013). The assembly and function of mitochondrial OXPHOS are dependent on a great number of proteins, mainly encoded by nuclear DNA and by mitochondrial DNA. Genetic defects in some of these proteins lead to various phenotypes with different ages of onset and course of the disease. Next-generation sequencing has significantly broadened our understanding of the genetic causes of mitochondrial disease (Jin et al., 2019; Li et al., 2019; Shen and Shi, 2019). In recent years, an increasing number of genes have been related to Alpers’ syndrome, many pathogenic mutations in the mitochondrial aminoacyl-tRNA synthetases (mt-aaRSs), such as *FARS2*, *CARS2*, *NARS2*, and *PARS2*, etc, are a novel cause of mitochondrial translation disorder, leading to Alpers’ syndrome (Elo et al., 2012; Sofou et al., 2015; Walker et al., 2016; Samanta et al., 2018; Sofou et al., 2021). This study demonstrated the pathogenic mechanism of gene variants resulting in the loss of receptor function (Nishikawa et al., 2020). The patients in this study exhibited different genotypes of Alpers’ syndrome, which suggests different genetic variations resulted in significant changes in mitochondrial function. In 2020, WES revealed a *de novo* missense variant of *GABRB2* (NM_021911.2: c.784G>T, p[Val262Phe]) in a child presenting with daily MSE and RHADS, that was associated with Alpers’ syndrome phenotype. However, the *GABRB2* gene encodes γ-GABA receptors. At present, the relationship between γ-GABA receptors and mitochondrial function remains unclear, and further research will be of importance.

The relationship between clinical phenotypes and genotypes has been studied thoroughly based on the collected results of genetic mutation variants. The clinical phenotypes of Alpers’ syndrome are variable, even in those with identical mutations, and this includes different degrees of neurological symptoms and hepatic dysfunction (Wu et al., 2018). The Alpers’ syndrome caused by *POLG* gene mutations usually affects children aged less than 4 years (Hannah and Yasir, 2021), and inevitably leads to death. Refractory epilepsy is a major phenotypic feature leading to the poor prognostic range and rapidly progressive disease. In the early stages of the disease, epileptic seizures are the most common. The epileptic seizures develop in the form of focal, multifocal or myoclonic seizures evolving into EPC or MSE. In this study, all the patients had exhibited epileptic seizures, most had a focal motor seizure and epileptic status (Supplementary Table S1). In this study, most seizures captured by EEG monitoring originated from the occipital lobe, which appears to be the main site for seizure genesis in Alpers’ syndrome. Numerous clinical and neuropathological studies revealed that the brain lobe is the key spot of the main area of neuronal degeneration and late post-stroke seizures in patients (Montine et al., 1995; Engelsen et al., 2008; Khan et al., 2012; Anagnostou et al., 2016; Wu et al., 2018). Recently, a complete study suggested interneuron and Purkinje cell pathology may be the mechanisms underlying seizure generation and maintenance (Hayhurst et al., 2019). Development is another common neurological feature, this can occur at any time in childhood and may reflect the nature of the process (Supplementary Table S1). In the course of the disease, the individuals with Alpers’ syndrome show different degrees of developmental delay, these findings in this study were consistent with those in previous studies (Supplementary Table S1). Liver involvement is the main characteristic of Alpers’ syndrome, which is rarely the presenting symptom and can rapidly progress to end-stage liver failure. Sodium valproate accelerates liver dysfunction and invariably ends in a fatality (Park et al., 2017). In the current study, four patients presented with progressive liver failure (Supplementary Table S1). In addition, sometimes viral prodrome symptoms, headaches, and vomiting can be observed, which may give rise to clinical suspicion of encephalitis (Rahman and Copeland, 2019), expanding the phenotypic spectrum of Alpers’ syndrome (Lim and Thomas, 2020). In recent years, with the advancement of genetic testing technology, Alpers syndrome caused by NON-*POLG* genes has also been continuously reported, including mitochondrial aminoacyl transfer RNA synthetase related genes (*PARS2, CARS2, FARS2 and NARS2*) and γ-GABA receptor related genes Gene (*GABRB2*). This study retrospectively compared the Alpers’ syndrome caused by NON-*POLG* and *POLG* gene mutations, and found that NON-*POLG* onset was earlier, mostly in the early infantile period, with prominent infantile spasms, less liver damage, and some microcephaly, most of the survival time after treatment is longer (Table 2; Figure 4), the phenotype is significantly different from the Alpers’ syndrome caused by *POLG* gene mutation (Sofou et al., 2015), so It may be better for these patients with mutations in NON-*POLG* to be diagnosed as Alpers-like syndrome. Differences in clinical phenotypes between the two groups may be related to the genes themselves, this aspect requires further research for clarification (Bao et al., 2019).

Our study systematically describes the EEG findings of various genotypes of Alpers’ syndrome. In this study, background slowing was more commonly seen in Alpers’ syndrome. Eighteen patients exhibited RHADS in the early stage of the disease, although six cases of RHADS on EEG performed nonspecific abnormalities during the late stage of the disease. RHADS may be associated with the staging phase (Boyd et al., 1986; Wolf et al., 2009; van Westrhenen et al., 2018). Our study is in agreement with the previous research that RHADS are highly distinctive in patients with Alpers’ syndrome. RHADS were all in the early stage of the disease with the association of epileptic status and no traces of the influence of drug treatment (Boyd et al., 1986; Wolf et al., 2009; van Westrhenen et al., 2018). In addition, a notable finding was that the patient suffered from RHADS with gamma oscillations (Figure 3). This spatial overlap suggests between the brain areas generating spikes and fast oscillations involve similar ‘‘hyperactive’’ neuronal networks. In this study, RHADS were based on focal abnormality along with epileptic discharges, however, the gamma oscillations were described in four patients in our series, this finding demonstrated that spikes are more sensitive. Yet, it is important to note that gamma activity more specific and accurate to determine the regions of the brain participating in seizure generation, and the occurrence of the fast oscillations directly reflects the degree of epileptic activity of the EEG (Melani et al., 2013). This finding supported the idea that RHADS is a kind of epileptic phenomenon (Zijlmans et al., 2012). We also found that patients with Alpers’ syndrome may have a high frequency of epileptic status. All patients experienced the same episodes of seizures and an indistinguishable EEG pattern during the early phase of the syndrome. EEG is an important tool for the examination of the diagnosis and prognosis of Alpers’ syndrome because the EEG patterns are consistent with fluctuations in the electrical properties of the brain. The EEG correlations at different stages of the disease not only help to recognize the clinical course of the disease, but also provide a platform for better utilization of EEG in the diagnosis of clinical disease development. The explicit EEG pattern remains the foundation for the diagnosis and may help to comprehend the mechanism of Alpers’ syndrome. However, it’s worth noting that Alpers’ syndrome caused by different genotypes showed similar EEG patterns in our study, which may be due to the involvement of a common electrophysiological mechanism. Therefore, future studies may be focused on applying digital video electroencephalography with high sampling rates and series of EEG patterns and the development of an automatic gamma oscillation analysis system.

To sum up, in this article, we the first integrated analysis of the EEG characteristics and clinical phenotype of patients with Alpers’ syndrome of various genotypes. We found that the patients having different pathogenic variants exhibited comparable phenotypes. There are certain differences in the clinical phenotype of Alpers’ syndrome caused by *POLG* gene mutations and NON-*POLG* gene mutations. and the differences in clinical phenotypes may be related to the genes themselves. So we believe that the clinical phenotype of NON-*POLG* gene mutations may be more appropriate to define Alpers-like syndrome. Due to our limited sample size, in the future, we will continue to collect samples and expand the sample size for further research. In addition, we defined EEG characteristics found specifically in Alpers’ syndrome. RHADS is a characteristic EEG finding in the early stage of Alpers’ syndrome, which is a kind of epileptic phenomenon, can provide clues for the early diagnosis of the disease. EEG characteristics of Alpers’ syndrome with various genotypes show similar EEG patterns, the reason may be related to the common electrophysiological mechanism. We only have a few cases and have yet to draw a definitive conclusion in terms of the relationship between pathogenic genes and Alpers syndrome. This is a summative retrospective study, but it will provide useful information for a deeper understanding of the pathophysiology of Alpers’ syndrome.

## Data Availability

The datasets presented in this study can be found in online repositories. The names of the repository/repositories and accession number(s) can be found in the article/Supplementary Material.

## Abbreviations

Bg, background; BS, burst suppression pattern; D, damaging; DC, Disease causing; DFP, disease-associated polymorphism with supporting functional evidence; DM, disease-causing mutation; EEG, Electroencephalogram; EPC, epilepsia partialis continua; FED, focal epileptiform discharges; FS, focal seizure; MFCS, Multifocal clonic seizures; MFED, multifocal epileptiform discharges; MS, myoclonic seizure; GTCS, generalized tonic-clonic seizures; MLH, migraine-like headache; GSW, generalised spike-wave or poly spike-wave discharges; Hyps, hypsarrhythmia; GS, generalized seizure; MFMS, multifocal myoclonus seizure; TS, tonic seizure; IS, infantile spasms; LP, likely pathogenic; P, pathogenic; NA, not available; PD, probably damaging; RHADS, rhythmic high-amplitude delta with superimposed (poly) spikes; SE, status epilepticus; VUS, uncertain significance.
