# Supplementary material for: Clinical Attributes and Electroencephalogram Analysis of Patients With Varying Alpers’ Syndrome Genotypes
Source: Front Pharmacol. 2021 Oct 6;12:669516. doi: 10.3389/fphar.2021.669516 (PMC8526534; doi:10.3389/fphar.2021.669516)
Supplement: Supplementary file 1 [file Table1.docx]

**TABLE S1: Clinical characteristics of patients with Alpers^’^ syndrome**

| Case sequence (reference number) | Sex | Age at onset | Initial symptoms | Age at seizure onset | Seizure types at onset | Courses of seizure | Seizure outcome | Development | Other features | EEG feature | Age at death |
| --- | --- | --- | --- | --- | --- | --- | --- | --- | --- | --- | --- |
| 1(The present case) | F | 2 y 8 m | Seizures | 2 y 8 m | SE | FS, SE | Refractory | Delayed | Hepatic dysfunction, hypoglycaemias, myasthenia, dysphagia | Slow bg, RHADS, MFED, FED | 1 y |
| 2(The present case) | M | 8 m | Seizures | 8 m | FS | FS | Refractory | Delayed | Hepatic dysfunction, hypotonia, myasthenia, dysphagia | FED, Slow bg, | 2 y 9 m |
| 3(The present case) | F | 3 y 11 m | Seizures | 3 y 11 m | SE | SE, FS | Refractory | Delayed | Hepatic dysfunction, hypotonia, vomiting, myasthenia, ataxia | Slow bg, RHADS, MFED, FED | Alive (7 y 3 m) |
| 4(The present case) | M | 2 m | DR | 1 y 10 m | SE | SE, FS | Refractory | Delayed | Fever, hepatic dysfunction, myasthenia | Slow bg, RHADS | 1 y 11 m |
| 5(The present case) | M | 3 m | Seizures | 3 m | SE | MS, SE | Refractory | Delayed | Vomiting | FED, Slow bg, RHADS | 1 y |
| 6(The present case) | F | 6 m | DR | 9 m | FS | FS, EPC | Refractory | Delayed | Gastrointestinal dysfunction, hypotonia, myasthenia | Slow bg, RHADS, FED | 1 y 2 m |
| 7(The present case) | M | 2 y | Seizures | 2 y | FS | FS, SE | Refractory | Delayed | Ataxia | FED, Slow bg, | Alive (4 y 7 m) |
| 8 (wiltshire et al., 2008) | F | Initially | Seizures | 5 y | FS | FS, SE | Refractory | Delayed | Visual aura, vomiting, memory impairment ,hemiparesis | Slow bg, RHADS, FED | 17 y |
| 9 (Wolf et al., 2009) | M | 6 y 7 m | Headache and vomiting | 6 y 7 m | FS | FS, SE, EPC, MFCS, MS | Refractory | Delayed | Lost speech and visual contact, developed optic atrophy, hepatic dysfunction | RHADS | 7 y 9 m |
| 10(Wolf et al., 2009) | F | 10 y 8 m | Headache and vomiting | 10 y 8 m | FS | FS, EPC | Refractory | Delayed | Hemiparesis, aphasia, lost visual contact | RHADS | 11 y 6 m |
| 11(Wolf et al., 2009) | F | 2 y 8 m | Seizures | 2 y 8 m | SE | SE, EPC, FS | Refractory | Delayed | Headache, vomiting, consciousness, stable, lost speech and visual contact | RHADS | Alive (6 y) |
| 12(Wolf et al., 2009) | F | 7 m | Seizures | 7 m | SE | SE | Refractory | Infantile scoliosis | Hypotonia and feeding difficulties , vomiting and pneumonia | NA | 10 m |
| 13(Wolf et al., 2009) | M | 9 y 3 m | Headache and visual symptoms | 9 y 3 m | SE | FS | Refractory | Delayed | Language delayed, visual aura, vomiting | RHADS | Alive (11 y) |
| 14 (Uusimaa et al., 2008) | F | 14 y | Visual symptoms | 14 y 11 m | GTCS | GTCS, SE | Refractory | NA | Headache, visual disturbance, somnolent, hepatic dysfunction | FED | 15 y |
| 15 (Uusimaa  et al., 2008) | F | 17 y | Seizures, | 17 y | FS, GTCS | FS, GTCS, SE | Refractory | NA | Visual symptoms, MLH, hepatic dysfunction | FED, | Alive (21 y) |
| 16 (Uusimaa et al., 2008) | F | 15 y | MLH | 15 y | FS, GS | FS, GS | Refractory | NA | Nystagmus, hepatic dysfunction | FED, Slow bg, GSW | 20 y |
| 17(Mccoy et al., 2011) | M | 17 m | Seizures | 17 m | EPC | EPC,FS | Refractory | Delayed | Multi-organ failure | Slow bg, RHADS, BS | 17 m |
| 18(Mccoy et al., 2011) | F | 10 m | Seizures | 10 m | FS | FS | Refractory | Delayed | Quadriplegia and brainstem dysfunction | Slow bg, RHADS | 12 m |
| 19(Mccoy et al., 2011) | F | 21 m | Seizures | 21 m | EPC | EPC | Refractory | Delayed | Coma, hypoglycaemia, tachycardia, oliguria | Slow bg, RHADS, | 42 m |
| 20(Mccoy et al., 2011) | F | 3 y 7 m | Seizures | 3 y 7 m | EPC | EPC | Seizures controlled | Congenital ataxia | Lethargy and vomiting | Slow bg, RHADS | Alive (3 y 7 m) |
| 21(Allen et al., 2014) | M | 10 m | Seizures | 10 m | EPC | EPC | Refractory | Delayed | Hypotonia | Slow bg, RHADS | 12 m |
| 22 (London et al., 2017) | F | 15 y 9 m | Headaches with visual symptoms | 16 y | SE | SE, MFMS, SE | Refractory | Delayed | Hepatic dysfunction, hypoglycaemia, tetraparesis, hypotonia, cognitive impairment | RHADS | Alive（17 y) |
| 23(Sofou et al., 2015) | M | Nb | DR | 7 m | SZ | MS, TS, Atypical S | Refractory | Delayed | Feeding difficulties, hypotonia, spastic tetraparesis, Scoliosis, Microcephaly, Visual impairment | FED | 16 y |
| 24(Sofou et al., 2015) | M | 2.5 m | Seizures, feeding difficulties | 2.5 m | FS | FS, IS | Refractory | Delayed | Hypotonia, dystonia, Microcephaly, Cortical visual impairment | FED, Hyps | 2 y 2 m |
| 25 (Samanta et al., 2018) | F | infant | Seizures | infant | SE | SE, FS | Refractory | Delayed | Dysphagia, autism spectrum disorder, hepatic dysfunction | FED | 13 y 4 m |
| 26 (Nishikawa et al., 2020) | M | 1 m | Hypertonia, poor feeding | 4 m | MS | MS, EPC | Refractory | Delayed | Hypotonia with mild spasticity | RHADS, BS | Alive (10 y 8 m ) |
| 27(Sofou et al., 2021) | M | at birth | Central hypotonia and feeding difficulties | 6 m | SE | GTCS, MS, EPC | Refractory | Delayed | Visual symptoms, Microcephaly, Hepatic dysfunction, Muscular weakness, Spastic quadriplegia | FED, MFED, Slow bg | 6 y |
| 28(Sofou et al., 2021) | M | 5 m | Seizures | 5 m | SE | GTCS, MS | Refractory | Delayed | Hypotonia, feeding difficulties, hepatic dysfunction, muscular weakness, spastic quadriplegia, sensorineural hearing impairment | NA | Alive (25 y) |
| 29(Walker et al., 2016) | F | infant | Motor and speech delays | 8 y | GTCS | GTCS, FS, EPC | Refractory | Motor and speech delays | Likely pneumonia and urinary tract infections | Slow bg, FED, GSW | 15 y |

Abbreviations: F, female; M, male; DR, developmental regression; SZ, seizure; FS, focal seizure; SE, status epilepticus; EPC, epilepsia partialis continua; MS, myoclonic seizure; GS, generalized seizure; TS,tonic seizure; AS, atypical absence seizure; IS, infantile spasms; GTCS, generalized tonic-clonic seizures; MLH, migraine-like headache; MFCS, Multifocal clonic seizures; MFMS, multifocal myoclonus seizure; Nb, newborn; NA, not applicable; GSW, generalised spike-wave or poly spike-wave discharges; FED,focal epileptiform discharges; MFED, multifocal epileptiform discharges; bg, background; RHADS, rhythmic high-amplitude delta with superimposed (poly) spikes; Hyps, hypsarrhythmia; BS, burst suppression pattern; MSE, rhythmic myoclonus status epilepticus
